# Targeting SARS-CoV-2 Polymerase with New Nucleoside Analogues

**DOI:** 10.3390/molecules26113461

**Published:** 2021-06-07

**Authors:** Vasiliki Daikopoulou, Panagiotis Apostolou, Sofia Mourati, Ioanna Vlachou, Maria Gougousi, Ioannis Papasotiriou

**Affiliations:** 1 Research Genetic Cancer Centre S.A. Industrial Area of Florina, GR53100 Florina, Greece; daikopoulou.vasiliki@rgcc-genlab.com (V.D.); apostolou.panagiotis@rgcc-genlab.com (P.A.); mourati.sofia@rgcc-genlab.com (S.M.); vlachou.ioanna@rgcc-genlab.com (I.V.); gougousi.maria@rgcc-genlab.com (M.G.); 2 Research Genetic Cancer Centre International GmbH, Baarerstrasse, 95, 6301 Zug, Switzerland

**Keywords:** nucleoside derivatives, COVID-19, quinazoline moiety

## Abstract

Despite the fact that COVID-19 vaccines are already available on the market, there have not been any effective FDA-approved drugs to treat this disease. There are several already known drugs that through drug repositioning have shown an inhibitory activity against SARS-CoV-2 RNA-dependent RNA polymerase. These drugs are included in the family of nucleoside analogues. In our efforts, we synthesized a group of new nucleoside analogues, which are modified at the sugar moiety that is replaced by a quinazoline entity. Different nucleobase derivatives are used in order to increase the inhibition. Five new nucleoside analogues were evaluated with in vitro assays for targeting polymerase of SARS-CoV-2.

## 1. Introduction

The ongoing pandemic COVID-19 has already infected more than 142 million people worldwide resulting in over 3 million reported deaths, with significant social and economic repercussions [1]. This disease is caused by severe acute respiratory syndrome coronavirus (SARS-CoV-2), there is no specific drug for inhibiting the replication of this virus. Coronaviruses are a large family of single-stranded RNA viruses (+ssRNA) that infect different animal species [2,3]. The SARS-CoV-2 RNA-dependent RNA polymerase (RdRp) is responsible for the replication of the virus. It can cause sickness with symptoms ranging from a common cold to severe symptoms and death. Their positive sense RNA acts like a messenger RNA (mRNA) and can be directly translated. However, these viruses encode for RNA-dependent RNA polymerases in order to accomplish their replication, by synthesizing a negative sense RNA strand which then acts as the template for the positive sense strand synthesis. Therefore, RdRp could be used as a potential drug target [4].

**Figure 1:**
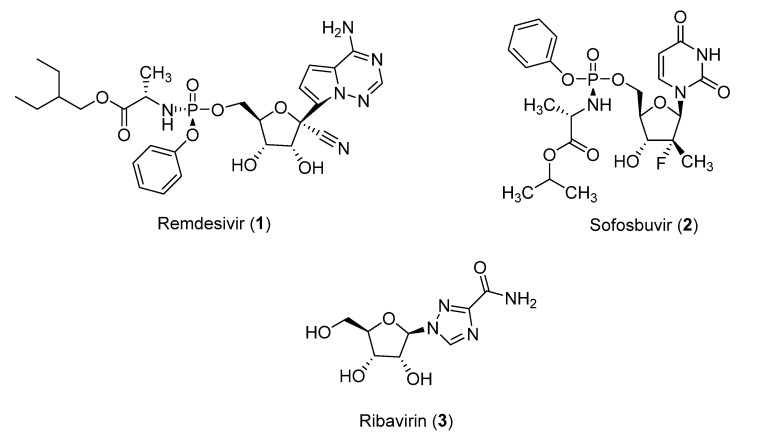
Remdesivir (**1**), Sofosbuvir (**2**) and Ribavirin (**3**).

There are several ways to inhibit the replication process of the virus. The first is through the inhibition of the polymerase itself; therefore, no replication is taking place. In another way, specific nucleoside analogues are used, which in turn does not allow the polymerization of the viral RNA. Nucleoside analogues are modified, and upon being incorporated to the nucleotide chain, they cannot form bonds with the next-added nucleotides, terminating the reaction [5]. In general, nucleoside analogue inhibitors lack the 3′-OH group, avoiding the formation of the 3′-5′ phosphodiester bond. Such inhibitors have been widely used both for DNA as well as for RNA polymerases [6,7]. The coronavirus RdRp could be targeted by the biggest class of small molecule-based antivirals which is the nucleoside analogues [8,9,10]. In recent studies many nucleoside analogues have been reported as candidate inhibitors of the SARS-CoV-2 RdRp [11,12]. Remdesivir (**1**) [13,14,15], Sofosbuvir (**2**) [16] and Ribavirin (**3**) [17,18] (Figure 1) have been evaluated against RdRp. Emtricitabine combined with Tenofovir, that are also known nucleoside analogues were assessed for their antiviral activity [19]. In fact, clinical trials have been designed to start using this combination of drugs in patients [20,21]. Furthermore, it is important to highlight that besides targeting RdRp, several recent proposals have been published for the treatment of SARS-CoV-2 targeting the SARS-CoV-2 Main Protease [22,23].

**Figure 2:**
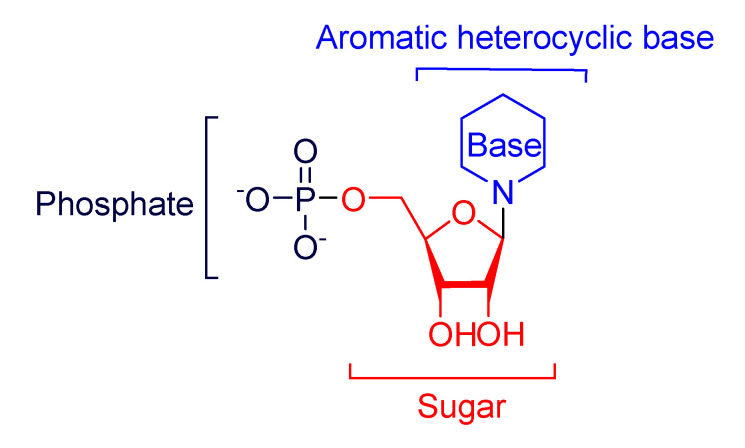
Areas for modification on nucleoside analogues.

Nucleoside analogue inhibitors are chemically synthesized derivatives of a nucleobase conjugated with a sugar moiety in which either the heterocyclic ring or the sugar moiety has been modified (Figure 2). Nucleoside analogues could be used as anticancer agents [24,25,26], as antibacterial agents [27,28] and as antiviral agents [29,30].

Previously, we reported the conjugation of an adenine with a quinazoline moiety [31]. Quinazolines are heterocyclic compounds and they have been investigated for their therapeutic potential. They have been evaluated as antimicrobial, anti-inflammatory and as anticancer agents and it has been proved that they play a significant role in medicinal chemistry [32,33,34]. In this study we synthesized nucleoside analogues which consist of purine and pyrimidine derivatives and a quinazoline moiety. Next, we aimed to identify whether the different analogues that have been designed and synthesized in our laboratory, could affect the SARS-CoV-2 polymerase, and particularly the catalytic subunit NSP12. The evaluation of the nucleosides we usedon in vitro assays, based on synthesis of DNA or RNA, using RNA as a template, and NSP12, as well as commercial RNA-dependent RNA polymerases.

## 2. Results

### 2.1. Synthesis of Modified Nucleoside Analogues

**Figure 3:**
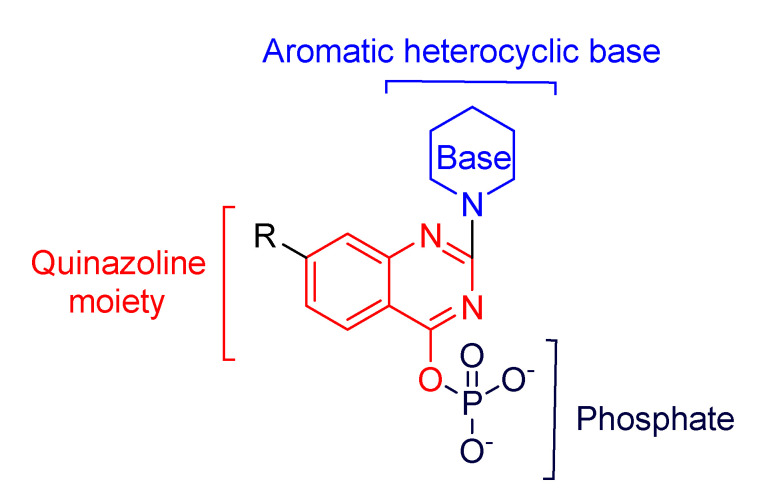
Replacement of sugar moiety with a quinazoline moiety for synthesis of new nucleoside analogues.

Our approach to synthesize a new group of nucleoside analogues was based on the modification of the sugar moiety into a quinazoline moiety (Figure 3). Nucleoside derivatives constitute a large class of antiviral agents and quinazoline analogues are molecules that have shown antiviral activity as well [35,36].

**Figure 4:**
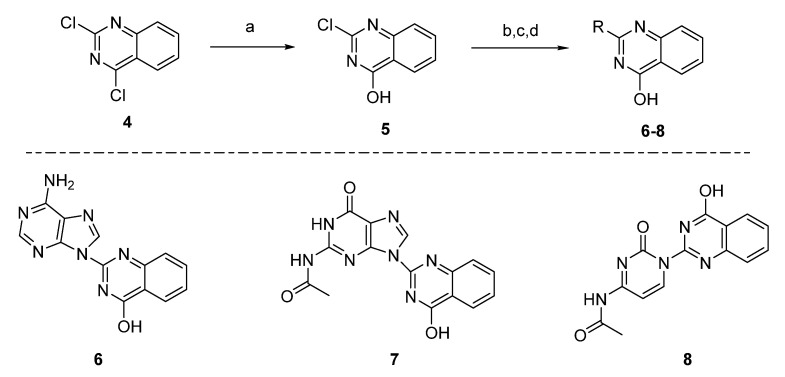
Synthesis of nucleoside analogues **6**–**8**. Reagents and conditions: (a) sodium hydroxide solution (2% NaOH), r.t. 4 h, (b) for compound **6**, adenine, cesium carbonate (Cs_2_CO_3_), silica gel, inert atmosphere, 130 °C, 3.5 h, (c) for compound **7**, *N*-acetyl guanine, cesium carbonate (Cs_2_CO_3_), silica gel, inert atmosphere, 130 °C, 24 h, (d) for compound **8**, *N*-acetyl cytosine, cesium carbonate (Cs_2_CO_3_), silica gel, inert atmosphere, 130 °C, 24 h.

The selective hydrolysis of the commercially available material **4**, was performed according to the literature [37]. The conjugation of quinazoline **5** with different nucleobase derivatives was performed via nucleophilic aromatic substitution (SNAr). The compound **6** was synthesized by the coupling of an adenine with the quinazoline 5 under nitrogen atmosphere, adding material silica gel as support. The desired product was formed in 41% yield. In order to synthesize compounds **7** and **8**, N-acetyl guanine [38] and N-acetyl cytosine [39] were synthesized accordingly, with already known procedures. The protection of these two molecules was performed to be directed to the formation of the specific N-C bond. The synthesis of compound **7** was performed with a yield of 37% and that of the compound **8** with a yield of 43% (Figure 4).

**Figure 5:**
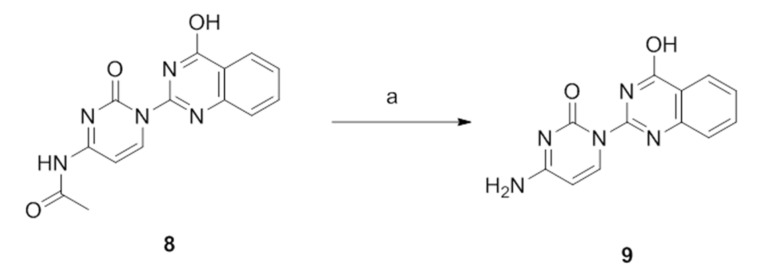
Deprotection of compound **8**. Reagents and conditions: (a) methylamine solution (40%), 65 °C, 24 h.

A deprotection step was performed in compound **8** in order to remove the acetyl group and receive the compound **9** in 82% yield (Figure 5).

**Figure 6:**
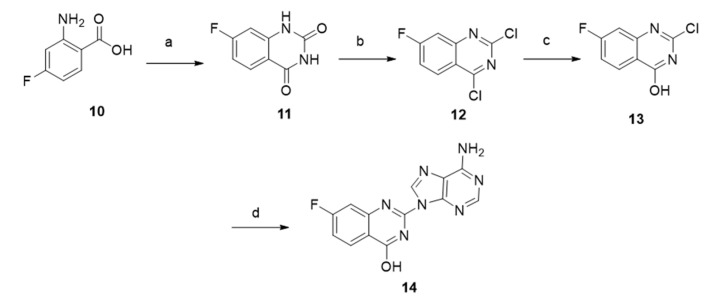
Synthesis of compound **14**. Reagents and conditions: (a) urea, 150 °C, 7 h, (b) POCl_3_, N, *N*-dimethylaniline, 110 °C, 7 h, (c) sodium hydroxide solution (NaOH 2%), 60 °C, 6 h, (d) adenine, cesium carbonate (Cs_2_CO_3_), silica gel, inert atmosphere, 130 °C, 24 h.

As we have already mentioned, the conjugation is achieved by regioselective N-arylation via nucleophilic aromatic substitution (SNAr). In order to evaluate whether a potential group on the quinazoline moiety will affect this step, we decided to insert a fluorine substituent to check if there is an increase of the yield. Since fluorine is considered an activated substrate for nucleophilic aromatic substitution, it was deliberately chosen. For this purpose, the compounds **11** and **12** were synthesized according to the literature. [40,41] Then, a selective hydrolysis of the quinazoline moiety was achieved and the compound **13** was formed in 55% yield. The conjugation of the adenine with compound **13** took place overnight and was formed in 53% yield. It is worth mentioning that in this experiment, we did receive higher yield than the yield in the conjugation without the fluorine substituent (Figure 6).

**Figure 7:**
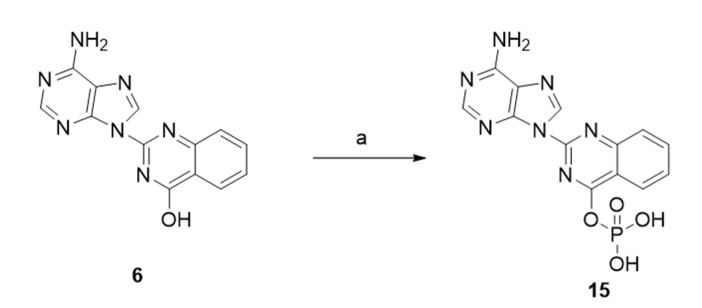
Phosphorylation of compound **6**. Reagents and conditions: (a) phosphorus pentachloride (P_2_O_5_), tetrabutylammonium bromide (TBAB), hydrogen peroxide solution (30% H_2_O_2_), CH_3_CN/H_2_O, 0 °C, 5 h.

According to the mechanism of action of a molecule during the inhibition of a polymerase, it is crucial that the molecule contains a hydroxyl or a phosphate group. Compounds **6**, **7**, **8** and **14** contain a hydroxyl group in the quinazoline moiety for this purpose. In order to evaluate a molecule which contains a phosphate group, we synthesized compound **15**. The phosphorylation was performed by using phosphorus pentoxide (Figure 7).

### 2.2. In Vitro Assays for Polymerase Inhibition

**Figure 8:**
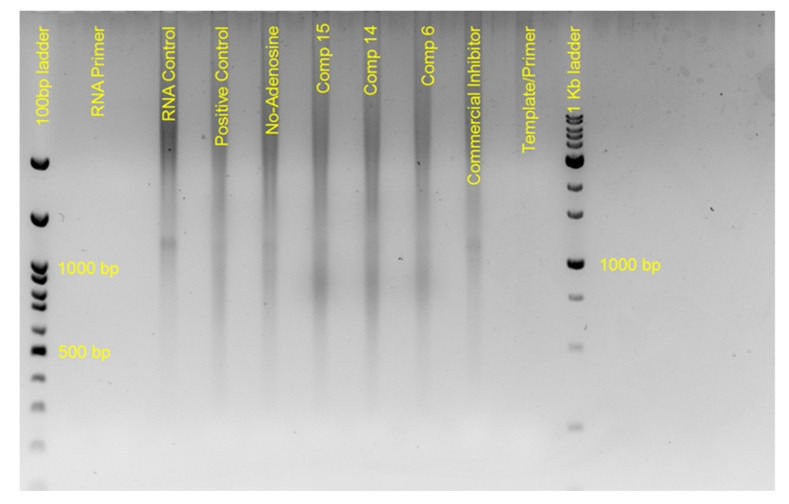
In-vitro assay using NSP12 polymerase and different adenine nucleoside inhibitors.

**Figure 9:**
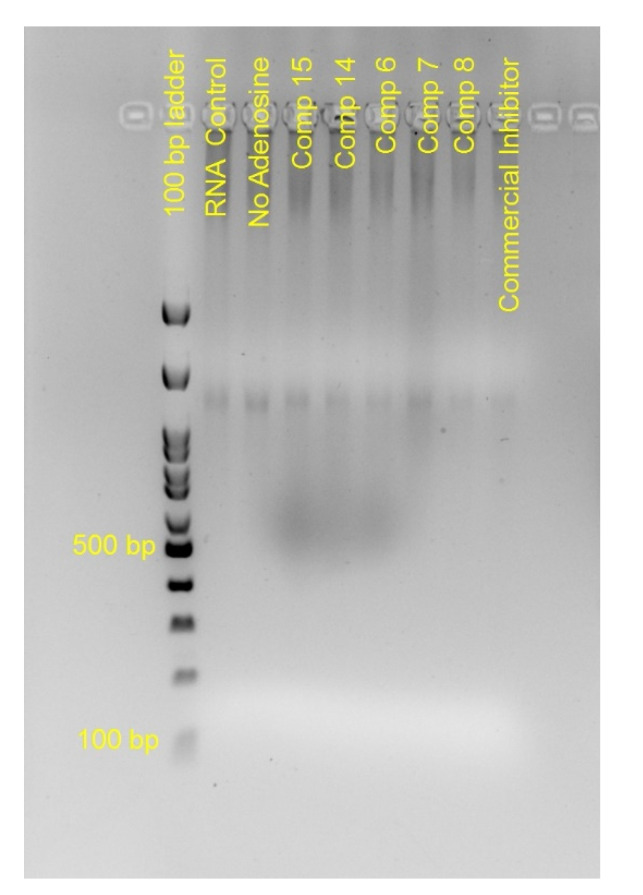
In-vitro assay using NSP12 polymerase and different adenine, guanine and cytosine nucleoside inhibitors.

The in-vitro results are presented in Figure 8 and Figure 9. In all reactions appropriate positive and negative controls were used. As positive control for the polymerase activity, we used the SARS-COV-2′s RNA, and non-modified nucleoside analogues, which was then polymerized by the commercial enzyme. Whether the polymerization takes place, the template is used, and other fragments are produced. Their lengths are not defined and vary, and thus give a smear between 600 and 1200 bp on the electrophoresis product. As a positive control for the inhibition assay, we used again SARS-COV-2′s RNA, the commercial enzyme and a mix of non-modified and commercial modified analogs (Cordycepin 5′-triphosphate sodium salt). Upon use of these analogues, the polymerization is inhibited and we observe on the Figure 8 a band, similar to the RNA itself. Additional negative controls included the use of rGTP, rCTP and rGTP but no rATP, which also gave an image similar to the RNA control, as it was expected, since no polymerization was performed, or it was performed until the NSP12 incorporated adenosine nucleosides. When we added our modified-analogues in the reactions we did not observe the same image as in the commercial inhibitor, indicating that no total inhibition took place. On the contrary, we did not observe the same image as in the positive control for polymerase, indicating that the polymerization could not be completed. The product in all substances was a smear between 700 and 900 bp. The smaller products probably indicate blocking of polymerization. The primers were not observed since they were smaller than the 100 bp of the ladder. We used the same data observed in Figure 9, both for adenine and also for the rest of the analogues.

## 3. Discussion

The aim of our study was to synthesize a new class of nucleoside analogues that could act as inhibitors of the SARS-CoV-2 polymerase. Compared to existing nucleoside analogues, the newly synthesized compounds were modified at the sugar moiety. The modification was performed by replacing the sugar entity with a quinazoline moiety. Initially, a nucleophilic aromatic substitution was performed in order to achieve the conjugation of the nucleobase with the quinazoline part. Different nucleobases were used in combination with quinazolines which are substituted with a hydroxyl group at the fourth position. In order to examine which parameters could affect the inhibition, a phosphorylation reaction was performed at the hydroxyl group of quinazoline. Additionally, the coupling of the adenine with the quinazoline which was substituted with fluorine at the seventh position was also achieved.

According to the literature, several infections which are caused by viruses such as human immunodeficiency virus (HIV), hepatitis V virus (HBV) or hepatitis C virus (HCV) can be treated with nucleoside analogues [42,43]. These agents are considered as safe and well tolerated since they are only used by the viral polymerase and not by human polymerase in DNA replication. Unfortunately, there has not been approved treatment for the coronavirus yet. Hence, in this work, we have specifically focused on inhibition of SARS-CoV-2 polymerase.

As a result of our efforts, we synthesized a new group of nucleoside derivatives which consist of adenine, cytosine and guanine moieties. In-vitro assays were performed for SARS-CoV-2 polymerase inhibition. The virus polymerase could ideally be studied in cells infected with viruses; however it’s not always feasible to do in-vitro assays with microorganisms, since specific facilities are required. On the other hand, different in-vitro assays have been proposed which do not require the cultivation of microorganisms. In all these, the polymerization of RNA is checked with the use of the catalytic subunit and appropriate nucleotides [44]. In our study we used an alternative of primer extension assay as described by Lu et al. [45]. The RNA template is polymerized using the RNA-dependent-RNA polymerase, when non-modified analogues are used, while the polymerization is blocked when modified nucleotides are incorporated into the reaction. The usage of our analogues on the assays, provided promising results, since inhibition of polymerization was observed, despite the fact that was not as strong as the inhibition caused by the commercial analogues. Finally, in further research, the compounds **6**, **7**, **8**, **14** and **15** will be inserted in liposomes in order to achieve the introduction of them inside the cell, as the results we presented in this work have encouraged us to make a drug delivery system to achieve our goal.

## 4. Materials and Methods

### 4.1. Chemistry

#### 4.1.1. General Information

Commercial reagents and anhydrous solvents were used without further purification. Flash chromatography was performed on the Reveleris^®^ X2 system, Buchi. ^1^H-NMR and ^13^C-NMR spectra were obtained using a Bruker Avance spectrometer at 400 MHz proton frequency (AV-III-HD, 400, Rheinstetten, Germany). All data were processed with TOPSPIN (version 3.5pl5, Bruker Biospin, Spring, TX, USA) software. Conventional abbreviations used for signal shape are as follows: s, singlet; d, doublet; dd, doublet of doublets; t, triplet; m, multiplet. LC-MS analysis (1260 Infinity Series HPLC, 6120 Quadrupole MSD, Agilent Technologies Inc.; Richardson, TX, USA) was carried out with the use of a reverse phase column, Zorbax RX-C8 (5 µm, 250 × 4.6 mm, Agilent Technologies, Santa Clara, CA, USA), maintained at 40 °C. Chromatograms were integrated and analyzed using OpenLAB Chemstation (version M8301AA, Revision C.01.07 Agilent Technologies Inc., Richardson, TX, USA). Mobile phase A was water, mobile phase B was acetonitrile and mobile phase C was glacial acetic acid. The mobile phases A, B and C were mixed at a ratio of 67.5:25.0:7.5 (*v*/*v*).

#### 4.1.2. Experimental Procedures and Analytical Data

*2-Chloroquinazolin-4-ol* (**4**). A round-bottom flask was charged with 2.4-dichloroquinazoline (2.5 g, 12.56 mmol) and with a solution of 2% sodium hydroxide (84 mL) and was stirred at room temperature for 4 h. The reaction mixture was filtered and then was acidified with glacial acetic acid. A white solid was formed. The solid was filtered and dried for 1 h at 65 °C to afford pure 2-chloroquinazolin-4-ol (**4**, yield: 96.2%, 2.17 g). ^1^H NMR (400 MHz, DMSO-*d*_6_) δ 13.26 (s, 1H), 8.10–8.09 (d, *J* = 8.0 Hz, 1H), 7.86–7.82 (t, *J* = 8.0 Hz, 1H), 7.62–7.60 (d, *J* = 8.0 Hz, 1H), 7.57–7.53 (t, *J* = 8.0 Hz, 1H). LC-MS (ESI): [M − H]^−^ = 179.

*2-(6-Amino-9H-purin-9-yl) quinazolin-4-ol* (**6**). To a two-neck round-bottom flask under inert atmosphere, a well-grounded mixture of adenine (1.3512 g, 10 mmol), 2-chloroquinazolin-4-ol (2.16 g, 12 mmol), cesium carbonate (3.2612 g, 10 mmol), silica gel (3.5 g) and 20 mL of dry DMSO were added. The solution was stirred at 130 °C for 3.5 h. Cold water (200 mL) was added in the reaction mixture. Then, it was filtered and dried over-night at 50 °C. The desired product was anchored on the silica gel. Methanol (200 mL) was added, and the solution was placed in the ultra-sonic bath. After 15 min, it was filtered under vacuum and was evaporated. The mixture was purified by flash chromatography (CHCl_3_/MeOH) in order to obtain the desired product 2-(6-amino-9*H*-purin-9-yl) quinazolin-4-ol (**6**, yield: 41%, 1.143 g). ^1^H NMR (400 MHz, DMSO-*d*_6_) δ 9.18 (s, 1H), 8.17 (s, 1H), 7.99–7.97 (d, *J* = 8.0 Hz, 1H), 7.57–7.52 (t, *J* = 8.0 Hz, 1H), 7.42–7.40 (d, *J* = 8.0 Hz, 1H), 7.22–7.18 (t, *J* = 8 Hz, 1H), ^13^C NMR (100 MHz, DMSO-*d*_6_) δ 170.91, 160.57, 153.82, 153.15, 152.90, 150.67, 144.00, 131.67, 126.08, 124.71, 122.75, 121.67, 109.53. LC-MS (ESI): [M − H]^−^ = 278.

*N-(9-(4-Hydroxyquinazolin-2-yl)-6-oxo-6,9-dihydro-1H-purin-2-yl)acetamide* (**7**). To a two-neck round-bottom flask under inert atmosphere, a well-grounded mixture of *N*-acetyl guanine (1.93 g, 10 mmol), 2-chloroquinazolin-4-ol (2.16 g, 12 mmol), cesium carbonate (3.2612 g, 10 mmol), silica gel (3.5 g) and 20 mL of dry DMSO were added. The solution was stirred at 130 °C for 24 h. Cold water (200 mL) was added in the reaction mixture. Then, it was filtered and dried over-night at 50 °C. Water ultra-pure (200 mL) was added to the solid and the solution was placed in the ultra-sonic bath. After 15 min, it was filtered under vacuum and was evaporated. The mixture was purified by flash chromatography (CHCl_3_/MeOH) in order to obtain the desired product *N*-(9-(4-hydroxyquinazolin-2-yl)-6-oxo-6,9-dihydro-1H-purin-2-yl)acetamide (**7**, yield: 37%, 1.247 g). ^1^H NMR (400 MHz, DMSO-*d*_6_) δ 9.86 (s, 1H), 9.03 (s, 1H), 8.05 (s, 1H), 7.70 (s, 1H), 7.54 (s, 1H), 7.36 (s, 1H), 2.20 (s, 3H), ^13^C NMR (100 MHz, DMSO-*d*_6_) δ 169.27, 164.89, 161.99, 161.36, 154.84, 149.16, 146.90, 141.50, 133.79, 126.17, 125.85, 124.59, 120.88, 109.41, 24.57. LC-MS (ESI): [M − H]^−^ = 336. Note: This compound is partially soluble in DMSO, which affects the NMR spectrum.

*N-(1-(4-Hydroxyquinazolin-2-yl)-2-oxo-1,2-dihydropyrimidin-4-yl) acetamide* (**8**). To a two-neck round-bottom flask under inert atmosphere, a well-grounded mixture of *N*-acetyl cytosine (1.53 g, 10 mmol), 2-chloroquinazolin-4-ol (2.16 g, 12 mmol), cesium carbonate (3.2612 g, 10 mmol), silica gel (3.5 g) and 20 mL of dry DMSO were added. The solution was stirred at 130 °C for 24 h. Cold water (200 mL) was added in the reaction mixture and was stirred for 20 min. After filtration under vacuum, a solution of HCl 1N was added and an off-white solid was precipitated. The solid was dried over-night at 50 °C in order to obtain the pure desired product *N*-(1-(4-hydroxyquinazolin-2-yl)-2-oxo-1,2-dihydropyrimidin-4-yl) acetamide (**8**, yield: 43%, 1.277 g). ^1^H NMR (400 MHz, DMSO-*d*_6_) δ 13.06 (s, 1H), 11.20 (s, 1H), 8.45–8.43 (d, *J* = 8Hz, 1H), 8.18–8.16 (d, *J* = 8Hz, 1H), 7.91–7.87 (t, *J* = 8Hz, 1H), 7.73–7.71 (d, *J* = 8Hz, 1H), 7.63–7.59 (t, *J* = 8Hz, 1H), 7.38–7.36 (d, *J* = 8Hz, 1H), 2.17 (s, 3H), ^13^C NMR (100 MHz, DMSO-*d*_6_) δ 171.32, 163.76, 161.45, 153.93, 147.55, 147.02, 145.80, 134.96, 127.51, 127.26, 126.17, 121.20, 96.42, 24.53. LC-MS (ESI): [M − H]^−^ = 296.

*4-Amino-1-(4-hydroxyquinazolin-2-yl)pyrimidin-2(1H)-one* (**9**). A round-bottom flask was charged with *N*-(1-(4-hydroxyquinazolin-2-yl)-2-oxo-1,2-dihydropyrimidin-4-yl)acetamide (**8**) (0.296 g, 1 mmol) and 1 mL of 40% methylamine solution. The reaction was stirred at 65 °C for 24 h. The mixture was evaporated, and then it was diluted with 10 mL of MeOH and was filtered. The solid was dried over-night for 24 h at 50 °C in order to obtain the desired product 4-amino-1-(4-hydroxyquinazolin-2-yl)pyrimidin-2(1H)-one (**9**, yield: 86%, 0.2193 g). ^1^H NMR (400 MHz, DMSO-*d*_6_) δ 13.43 (s, 1H), 8.39–8.37 (d, *J* = 8Hz, 1H), 8.13–8.11 (d, *J* = 8Hz, 1H), 7.90 (s, 2*H*), 7.86–7.82 (t, *J* = 7Hz, 1H), 7.65–7.63 (d, *J* = 8Hz, 1H), 7.54–7.50 (t, *J* = 7Hz, 1H), 6.05–6.03 (d, *J* = 8Hz, 1H), ^13^C NMR (100 MHz, DMSO-*d*_6_) δ 165.80, 160.95, 155.15, 147.79, 146.68, 140.93, 134.86, 126.45, 126.11, 120.55, 97.06. LC-MS (ESI): [M − H]^−^ = 254.

*7-Fluoroquinazoline-2,4(1H,3H)-dione* (**11**). A round-bottom flask was charged with 2-amino-benzoic acid (7.69 g, 50 mmol) and urea (6.84 g, 114 mmol) and was placed at 150 °C for 7 h. The mixture was cooled to room temperature and 500 mL of water was added. Then, it was stirred for 2 h. The mixture was filtered under vacuum. The solid was diluted again with 100 mL of water (×3), was filtered under vacuum and dried at 50 °C over-night, in order to obtain the desired product 7-fluoroquinazoline-2,4(1H,3H)-dione (**11**, yield: 72.8%, 6.652 g). ^1^H NMR (400 MHz, DMSO-*d*_6_) δ 11.29 (s, 2H), 7.96–7.92 (t, *J* = 9 Hz, 1H), 7.03–6.99 (t, *J* = 7 Hz, 1H), 6.90–6.87 (d, *J* = 10 Hz, 1H). LC-MS (ESI): [M − H]*^−^* = 179.

*2,4-Dichloro-7-fluoroquinazoline* (**12**). A round-bottom flask was charged with 7-fluoroquinazoline-2,4(1H,3H)-dione (0.715 g, 3 mmol), in POCl_3_ (5.57 mL, 59.8 mmol). Then, dropwise addition of N, N-dimethylaniline (0.76 mL, 1 equiv.) was performed. The mixture was heated at 110 °C and kept at reflux for 3 h. The reaction mixture was cooled to room temperature and was poured onto an ice-CH2Cl2 mixture. Then, 30 mL of CH_2_Cl_2_ were added and washed with 15 mL of water (×3). All the organic layers were dried over Na_2_SO_4_ and evaporated to obtain a green solid. The solid was purified with flash chromatography (hexane/ethyl acetate) to afford the pure 2,4-dichloro-7-fluoroquinazoline (**12**, yield: 73%, 0.4304 g) ^1^H NMR (400 MHz, DMSO-*d*_6_) δ 8.44–8.40 (dd, *J* = 6Hz), 7.95–7.91 (m, 1H), 7.86–7.81 (m, 1H). LC-MS (ESI): [M − H]^−^ = 215.

*2-Chloro-7-fluoroquinazolin-4-ol* (**13**). A round-bottom flask was charged with 2,4-dichloroquinazoline (0.864 g, 4 mmol) and with solution of 2% sodium hydroxide (26.8 mL) and was stirred at 60 °C for 6 h. The reaction mixture was filtered and then was acidified with glacial acetic acid. A white solid was formed. The solid was filtered and dried for 1 h at 65 °C to afford pure 22-chloro-7-fluoroquinazolin-4-ol (**13**, yield: 55%, 0.4362 g). ^1^H NMR (400 MHz, DMSO-*d*_6_) δ 13.38 (s, 1H), 8.16–8.12 (t, *J* = 8Hz, 1H), 7.44–7.38 (m, 2H). LC-MS (ESI): [M − H]^−^ = 197.

*2-(6-Amino-9H-purin-9-yl)-7-fluoroquinazolin-4-ol* (**14**). To a two-neck round-bottom flask under inert atmosphere, a well-grounded mixture of adenine (0.27 g, 2.0 mmol), 2-chloro-7-fluoroquinazolin-4-ol (0.4356 g, 2.2 mmol), cesium carbonate (0.656 g, 2 mmol), silica gel (0.70 g) and 2 mL of dry DMSO were added. The solution was stirred at 130 °C for 7 h. Cold water (20 mL) was added to the reaction mixture. Then, it was filtered and dried over-night at 50 °C. The desired product was anchored on the silica gel. Methanol (20 mL) was added, and the solution was placed in the ultra-sonic bath. After 15 min, it was filtered under vacuum and was evaporated. The mixture was purified by flash chromatography (CHCl_3_/MeOH) in order to obtain the desired product 2-(6-amino-9*H*-purin-9-yl)-7-fluoroquinazolin-4-ol (**14**, yield: 53%, 0.4362 g) ^1^H NMR (400 MHz, DMSO-*d*_6_) δ 9.13 (s, 1H), 8.28 (s, 1H), 8.12–8.10 (m, 1H), 7.28–7.26 (d, *J* = 8 Hz, 1H), 7.20–7.16 (m, 1H), ^13^C NMR (100 MHz, DMSO-*d*_6_) δ 167.76, 166.29, 163.83, 159.72, 152.84, 152.64, 151.70, 151.57, 144.69, 129.03, 128.92, 118.04, 112.75, 112.52, 110.07, 109.86, 109.25 LC-MS (ESI): [M − H]^−^ = 296.

*2-(6-Amino-9H-purin-9-yl) quinazolin-4-yl dihydrogen phosphate* (**15**). A round-bottom flask was charged with phosphorus pentachloride (0.5 mmol, 0.0694 g), 11 mL of CH_3_CN and 11 mL of H_2_O and was placed in an ice-bath. A dropwise addition of a solution 30% H_2_O_2_ was performed within 10 min and the solution was stirred for 5 min. 2-(6-amino-9H-purin-9-yl) quinazolin-4-ol (**6**, 0.279 g, 1 mmol) and tetrabutylammonium bromide (0.32 g, 1 mmol) were added to the flask and the reaction mixture was stirred for 5 h in the ice-bath. After completion of the reaction, the mixture was filtered and the solid was dried at 50 °C over-night. The solid was diluted to 20 mL MeOH and was stirred for 30 min. It was filtered and dried at 50 °C for 2 h to afford the desired product 2-(6-amino-9H-purin-9-yl) quinazolin-4-yl dihydrogen phosphate (**15**, yield: 18%, 0.0646 g). ^1^H NMR (400 MHz, DMSO-*d*_6_) δ 9.01 (s, 1H), 8.35, (s, 1*H*), 8.17–8.15 (d, *J* = 8Hz, 1H), 7.87–7.84 (t, *J* = 8Hz, 1H), 7.66–7.64 (d, *J* = 8Hz, 1H), 7.55–7.52 (t, *J* = 8Hz, 1H), ^13^C NMR (100 MHz, DMSO-*d*_6_) δ 167.77, 160.17, 153.28, 153.62, 149.21, 148.13, 144.61, 133.22, 126.12, 125.28, 124.56, 120.90, 109.28, 31P NMR (160 MHz, DMSO-*d*_6_) δ −1.09 [M + H]^+^ = 360.

### 4.2. In Vitro Assays

In the assays, the RdRp catalytic domain of RNA-directed RNA polymerase (Reference: PX-COV-P006, ProteoGenix, Schiltigheim, France) was used. As a template, we used the Genomic RNA from 2019 Novel Coronavirus, Strain: 2019-nCoV/USA-WA1/2020 (Reference: ATCC-VR-1986D, ATCC, Wessel, Germany), while primers (RNA or DNA) complementary to specific regions were obtained from Eurofins. In the reactions, we used the 10X First-Strand Buffer and RNase Inhibitor from the Amino Allyl MessageAmp™ II aRNA Amplification Kit (Reference: AM1753, Thermo Fisher Scientific, Bremen, Germany), as well as dNTPs/rNTPs. A commercial inhibitor Cordycepin 5′-triphosphate sodium salt (Reference: C9137, Sigma-Aldrich, Taufkirchen, Germany) was used as a positive control. The run conditions were 37 °C for 2 h, and samples were run on agarose gels.

The positive sample included everything mentioned above (without the inhibitor), as well as commercial nucleotides, to verify that the synthesis is performed without any problems, while a second positive control, including the commercial inhibitor, was used to compare our analogues and their ability to terminate the reaction. Negative controls, including only primer, or without specific nucleotides, were used as well. In the reactions, where our nucleoside analogues were added, commercial normal nucleotides were also added at a ratio of 1:1. The products were run on agarose gels using an appropriate ladder. Gels were stained with MidoriGreen Dye, which stains dsDNA, ssDNA, dsRNA and ssRNA.

## 5. Conclusions

In summary, a new class of nucleoside analogues were synthesized which contain a quinazoline moiety instead of a sugar entity. Five of these new compounds were tested in-vitro and based on the results, it was demonstrated that the addition of modified nucleoside analogues seems to inhibit the polymerization activity of SARS-CoV-2 RNA-dependent RNA polymerase.

## 6. Patents

All of these results have been filed for a PCT patent.

## Data Availability

Not applicable.
